# Complete Genome Sequences of BK Polyomavirus Strains from Two Patients with Urinary Tract Infection, Sequenced Using the Ion Torrent Platform

**DOI:** 10.1128/genomeA.01293-17

**Published:** 2017-11-16

**Authors:** Graham Rose, Kyriaki Ranellou, Raju Misra, Colin Crump, David Wooldridge, Surendra Parmar, Christopher Maddren, Saheer Gharbia, Hamid Jalal

**Affiliations:** a Genomics Research Unit, Public Health England, London, United Kingdom; b University of Cambridge, Department of Virology, Cambridge, United Kingdom; c PHE, Public Health Laboratory, Clinical Microbiology and Public Health Laboratory, Addenbrooke’s Hospital, Cambridge, United Kingdom; d Life Sciences Solutions, Thermo Fisher Scientific, Paisley, United Kingdom

## Abstract

BK polyomavirus is an important pathogen in kidney transplant patients. We report here two complete genome sequences, those of isolates CAMB-1035 and CAMB-1055, identified in two urine samples tested for urinary tract infection at a hospital in eastern England, United Kingdom. Variation and phylogenetic analyses indicate that both isolates belong to subtype Ib-1.

## GENOME ANNOUNCEMENT

BK polyomavirus (BKPyV) is a nonenveloped double-stranded DNA (dsDNA) virus and ubiquitous human pathogen, with up to 90% of adults worldwide being seropositive (1, 2). Primarily, infection occurs during childhood, after which a latent asymptomatic infection is established in the urogenital tract (3). However, in immunocompromised patients, such as bone marrow and renal transplant recipients, active infection is associated with a wide range of clinical manifestations, including serious damage to the bladder and kidneys (1, 3). Here, we report the complete genome sequences of BKPyV isolates from two patients, collected from residual urine samples destined for disposal after being sent for routine diagnosis at Public Health England (PHE) Cambridge for suspected urinary tract infection.

After recording available demographic and clinical data, samples were anonymized, and two of them, CAMB-1035 and CAMB-1055, were selected for genome sequencing after testing positive for BKPyV by an in-house quantitative PCR (qPCR) assay. DNA extraction and sequencing were performed as follows: nucleic acid was extracted using the PureLink viral RNA/DNA kit (Invitrogen) from 100 μl of urine, followed by OneStep PCR inhibitor removal column (Zymo Research). The qPCR indicated low viral concentrations (threshold cycle [*C_T_*], 26.7 and 27.5); therefore, a PCR amplification step with a set of six custom BKPyV-specific primers covering the complete genome was used. Libraries were prepared using the Ion Xpress Plus fragment library kit (Life Technologies), per the manufacturer’s instructions, and then size selected by 2% E-Gel SizeSelect (Invitrogen). Fragment size and concentration were measured by an Agilent Bioanalyzer and a Qubit fluorometer, respectively. Libraries were amplified on the One Touch 2 with the Ion PGM template OT2 400 kit (Life Technologies) and sequenced on an Ion Torrent PGM sequencer using the 318 Chip kit version 2 and Ion PGM Hi-Q sequencing kit (Life Technologies). Sequencing reads were trimmed by base quality and human host sequences filtered as described previously (4).

After assembly using metaSPAdes (version 3.7.1) (5), each BKPyV assembly consisted of a single circular contig which was split using Gepard version 1.40 (6) at the origin of replication based on the BKPyV-Dun reference (GenBank accession number NC_001538), generating a 5,141-bp genome. Using a pipeline with BWA (7) and SAMtools (8), 98,095 reads (20.0 Mb) and 158,009 reads (30.8 Mb) mapped to the assembled genomes of CAMB-1035 and CAMB-1055, respectively, both with 100% genome coverage, and at average 3,878× and 5,995× coverages, respectively.

Nearly 300 BKPyV strains have now been completely sequenced. BLASTn analysis of the two genomes showed >97% sequence identity to BKPyV-Dun and a characteristic genome organization of six genes encoding regulatory and structural proteins separated by a noncoding region. The highest sequence identity (>99.8%) was to isolates SJH-LG-152 (GenBank accession number JN192431) and SJH-LG-306 (GenBank accession number JN192437). Globally, there are four BKPyV subtypes (I to IV), with subtype I divided into four subgroups (9). Phylogenetic reconstruction by maximum likelihood using 33 genomes representative of the main lineages (10) clustered both genomes within subgroup Ib-1, which is within the most prevalent subtype (11). After filtering and annotating variants compared to BKPyV-Dun (12, 13), 15/79 CAMB-1035 single-nucleotide polymorphisms (SNPs) and 18/85 CAMB-1055 SNPs were nonsynonymous, of which most fell within the structural region encoding the capsid proteins and agnoprotein (26 nonsynonymous SNPs [78.8%]).

### Accession number(s).

All sequences are publicly available and can be found within the EBI European Nucleotide Archive under the chromosome accession numbers LT934539 (CAMB-1035) and LT960370 (CAMB-1055) and ENA study accession number PRJEB22539. The version described in this paper is the first version.
